# Investigation of transient eye closure evoked with bright light in the patients with intermittent exotropia

**DOI:** 10.1186/s12886-021-02046-7

**Published:** 2021-07-31

**Authors:** Won Jong Choi, Yeonji Jang, Seong-Joon Kim, Jae Ho Jung

**Affiliations:** 1 Department of Ophthalmology, Seoul National University Hospital and Seoul National University College of Medicine, Seoul, South Korea; 2 Department of Ophthalmology, Uijeongbu Eulji Medical Center and Eulji University, Uijeongbu, South Korea

**Keywords:** Eye closure, Intermittent exotropia, Photosensitivity, Photophobia, Squint

## Abstract

**Background:**

This study aimed to present a simple method for evaluating transient eye closure (TEC) evoked by bright light and find the agreement between TEC and photosensitivity. We also assessed the associated factors with TEC in the patients with intermittent exotropia (IXT).

**Methods:**

In this retrospective study, IXT patients were exposed to different brightness: darkness, low-intensity white light, and high-intensity white light using a near-infrared camera vision monitor system (Mon CV3, Metrovision, France). TEC was considered to be present if the subject closed his or her eyes immediately, and for more than half of the scotopic lid fissure distance in response to the high-intensity or low-intensity photopic stimulus of light, compared with lid fissure distance in the scotopic phase. We assessed the presence of photosensitivity using a questionnaire and evaluated the agreement between TEC and photosensitivity. We also investigated the sensory fusion, motor fusion, and pupil dynamic components for the existence of TEC in IXT patients.

**Results:**

Sixty-one patients with IXT were included. With the new method to evaluate TEC under different light intensities, 27 (44.3%) of the 61 IXT patients showed TEC, and 34 (55.7%) did not demonstrate TEC. TEC under high-intensity white light had a strong correlation with self-reporting photosensitivity (*r* = 0.77). The smaller angle of deviation at near was associated with the presence of TEC, with statistical significance (*p* = 0.04). Normal sensory status at a distance was significantly associated with TEC (*p* <  0.01). Multivariate analysis using multiple logistic regression analysis showed that normal sensory status was significantly associated with TEC (*p* = 0.02).

**Conclusions:**

The test using a near-infrared camera vision monitor system was a simple and objective tool in identifying TEC evoked by bright light. The presence of TEC strongly correlated with self-reporting photosensitivity in patients with IXT. However, TEC may be an independent phenomenon with motor alignment, stereopsis, and pupil reflex pathway in patients with IXT.

**Supplementary Information:**

The online version contains supplementary material available at 10.1186/s12886-021-02046-7.

## Background

Intermittent exotropia (IXT) is one of the most common types of childhood strabismus and occurs more frequently in Asian populations [1, 2]. Transient eye closure (TEC) can be a reason to present for ophthalmology evaluation, and exposure in the bright light usually the main trigger in this phenomenon in IXT patients. This symptom affects the patient’s health-related quality of life [3], and it often persists even after successful strabismus surgery [4]. Several studies have suggested that the mechanism of TEC is a decreased threshold of bright light, abnormal binocular summation, diplopia, or Fechner’s paradox, which is, in brief, the apparent increase in the brightness of a figure caused by closing one eye after viewing the figure with both eyes open [4–9]. Moreover, a recent study revealed that the reflexive eyelid movement is a naturally occurring response to light and is controlled by neural circuits that exist in the brainstem [10]. Photic blink reflex can function as an accessory pupil, further controlling retinal luminance in addition to pupil size [11]. Since this reflex has a shorter latency than the pupil light reflex, it may play a more significant role in modulating retinal luminance under both a light stimulus and a steady-state light [11, 12]. Even though the TEC is such a common feature in IXT, little is known about the association between TEC and IXT, and, thus, we should explore the phenomenon more thoroughly. We believe that the better understanding of TEC would bring us more information about the IXT. However, since there is no quantitative tool to evaluate eye closure in bright light, we can only depend on the subjective statement. Considering the frequency of the IXT patients’ symptom, we need a more objective and reproducible tool to evaluate TEC in a hospital setting.

We now report a simple method to more objectively assess TEC evoked under different light intensities using a near-infrared camera monitoring system. We evaluated the agreement between the presence of TEC under the new test method and self-reporting photosensitivity in patients with IXT. We also investigated the association factors related to TEC.

## Methods

This retrospective chart review study was conducted at Seoul National University Children’s Hospital between 2018 and 2019. The study was reviewed and approved by the Institutional Review Board of Seoul National University Hospital (2010–080-1164), Seoul, South Korea. All study procedures adhered to the tenets of the Declaration of Helsinki.

### Subjects

All the patients with childhood IXT without any intervention were included in the study. There was no age restriction. All subjects underwent a complete ophthalmologic examination and TEC testing under different light intensities using a near-infrared camera vision monitor system. In addition, we defined that a subject has photosensitivity if the patient answers ‘yes’ to the three out of five questions described in supplement 1.

The information about the onset of IXT was gathered from the parent’s report. Every procedure was done with his or her best-corrected visual acuities. Each patient underwent an alternate prism cover test to measure the angle of deviation at distance (5 m) and near (30 cm) the target, along with the evaluation of the office-based control scale (CS) for each eye. The control scale ranges from 0 (IXT best control) to 5 (constant exotropia) [13], and the control scale was divided into two groups for the analysis: 0–2 (well-controlled IXT), and 3–5 (poorly controlled IXT). Moreover, near (40 cm) stereoacuity was measured using the Fly Stereo Acuity Test with Lea symbols (Vision Assessment Co., Elk Grove Village, IL, USA) while wearing his or her best refractive correction. Worth’s Four Dot (W4D) test was also performed for each patient for the evaluation of sensory status. Subjects were excluded if they were diagnosed with: amblyopia, anisometropia greater than 1.5D, refractive error greater than 3.00 D, astigmatism greater than 1.50 D, a control score of 5 at a distance or near, vertical deviation of > 5 prism diopters, paralytic or restrictive esotropia, a known global developmental or neurological abnormality, or the inability to perform the near-infrared camera vision monitor system.

### Evaluation of transient eye closure under different light intensities

**Fig. 1:**
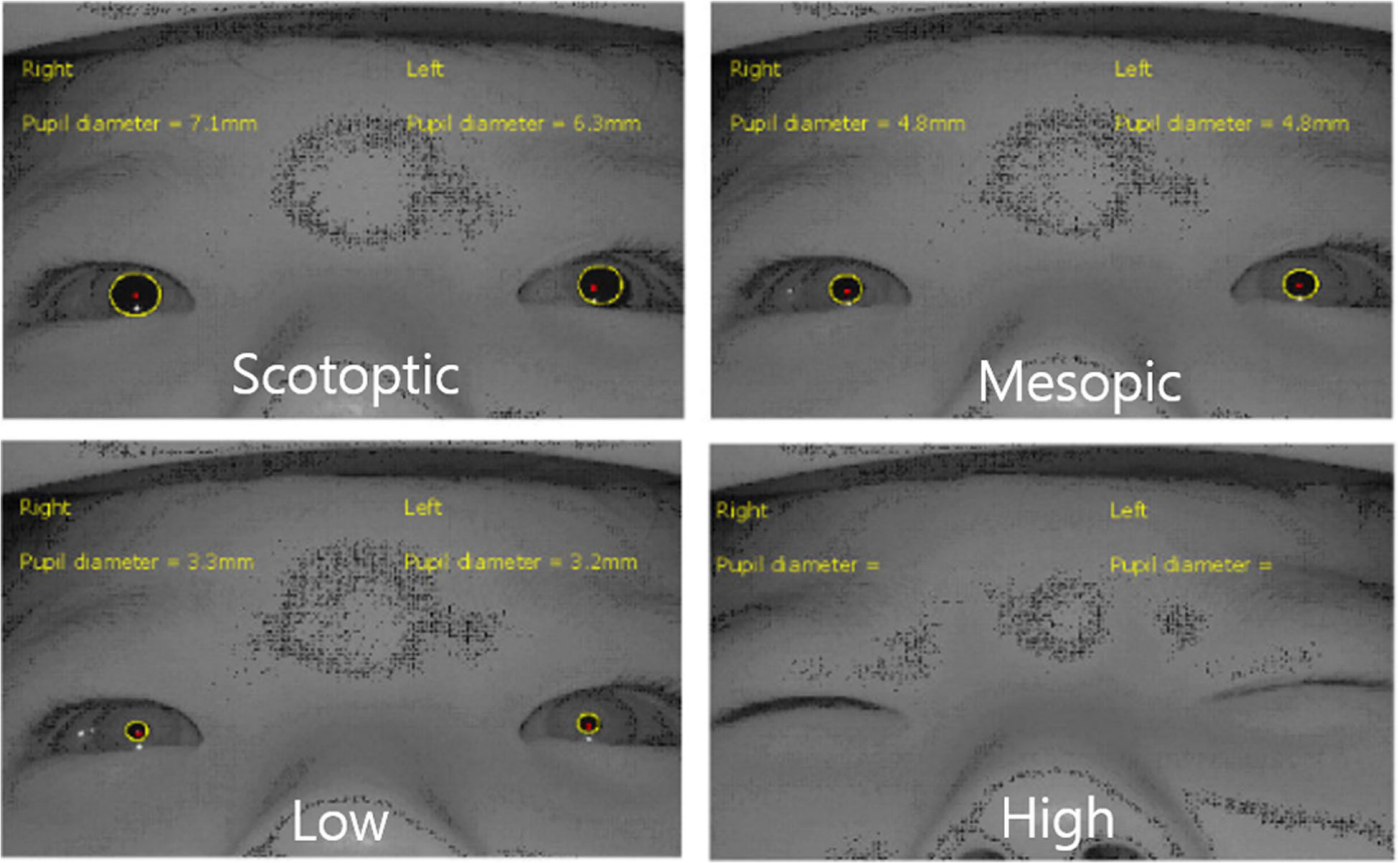
We captured snapshots of reflexive eye closure and pupil changes from a live video feed using a near-infrared vision monitoring system (Mon CV3, Metrovision, France) under different light intensities. Scotopic phase (darkness) for 3300 ms, mesopic phase (under room light without light stimulation) for 200 ms, scotopic phase for 3300 ms, low-intensity white light phase (10 cd/m^2^) for 200 ms, scotopic phase for 3300 ms, and high-intensity white light phase (100 cd/m^2^) for 200 ms.

We used a near-infrared camera vision monitor system (Mon CV3, Metrovision, France) to evaluate TEC. The patient was exposed to each phase as follows: scotopic phase (darkness) for 3300 ms, mesopic phase (under room light without light stimulation) for 200 ms, scotopic phase for 3300 ms, low-intensity white light phase (10 cd/m^2^) for 200 ms, and high-intensity white light phase (100 cd/m^2^) for 200 ms. The stimulator was equipped with near-infrared illumination (880 nm) and a high-resolution near-infrared camera that allowed for the measurement of pupil diameter even in complete darkness. Each subject maintained orthotropic throughout the test. Images of the eyes and eyelids were acquired and processed in real time (30 images per second). TEC was supposed to be present if the subject closed eyes more than half in response to light, compared with the one in the scotopic phase (Fig. 1). The software also provided in the pupillometry automatically outlined the pupillary contour on the images, ensuring the accuracy of the measurements (accuracy = 0.1 mm) under controlled illumination conditions. The presence of TEC was defined by a masked examiner (JHJ). TEC includes both monocular and binocular, and the examiner was masked to the presence of photosensitivity and TEC. The pupil dynamics were also acquired with eyelid position simultaneously.

### Statistical analysis

We analyzed the association between the TEC and self-reporting photosensitivity results using Pearson’s chi-square test and Phi correlation test. We used cross tab analysis to evaluate the relationship between sex, W4D test, photosensitivity, office-based control scale, and the TEC. To investigate the relationships between the onset of IXT, stereoacuity, dynamics of the pupil, and the TEC, we used a series of Student’s t-tests. Then, for the multivariate analysis, the associated factors were analyzed using a multivariate binomial logistic regression test. All analyses were performed using SPSS software version 23.0 (SPSS, Inc., Chicago, IL, USA).

## Result

Sixty-one patients with IXT were enrolled in this study. There were 26 females (42.6%) and 35 males (57.4%). The average age of the patients was 6.3 years. Other demographic features are shown in Table 1.

**Table 1:** Association between clinical characteristics and transient eye closure (TEC) in intermittent exotropia patients

	Overall (*n* = 61)	Transient eye closure (+) (*n* = 27)	Transient eye closure (−) (*n* = 34)	*p*-value	Multivariate analysis (*p-*value)
Onset of IXT (years,mean ± SD)	4.13 ± 2.13	4.07 ± 1.94	4.18 ± 2.29	0.85	0.11
Photo-sensitivity					
Yes	29 (47.5%)	23 (74.1%)	6 (26.5%)	< 0.01^a^	< 0.01^a^
No	32 (52.5%)	4 (25.9%)	28 (73.5%)		
Distance angle (PD,mean ± SD)	26.15 ± 6.39	24.41 ± 7.29	27.53 ± 5.28	0.06	0.99
Near angle (PD, mean ± SD)	24.67 ± 7.38	22.37 ± 7.87	26.50 ± 6.51	0.03^a^	0.10
Control scale					
0 ~ 2 (good-control)	49 (80.3%)	21 (77.8%)	28 (82.4%)	0.66	0.39
3 ~ 5 (poor-control)	12 (19.7%)	6 (22.2%)	6 (17.6%)		
Distance W4D					
Normal	32 (56.1%)	20 (76.9%)	12 (38.7%)	< 0.01^a^	0.02^a^
Abnormal	25 (43.9%)	6 (23.1%)	19 (61.3%)		
Near W4D					
Normal	43 (75.4%)	21 (80.8%)	22 (71.0%)	0.39	0.26
Abnormal	14 (24.6%)	5 (19.2%)	9 (29.0%)		
Near stereoacuity (arcsec, mean ± SD)	63.65 ± 75.32	75.42 ± 103.51	53.77 ± 37.95	0.32	0.17

^a^statistical significance, *W4D* Worth 4 dot test, *SD* Standard deviation

With the new method to evaluate TEC under different light intensities, 27 (44.3%) of the 61 IXT patients showed TEC, and 34 (55.7%) did not demonstrate TEC (Table 1). Among the 29 patients with photosensitivity, 23 patients (79.3%) had TEC and six patients (20.7%) were without TEC. Among the 32 patients without photosensitivity, four patients (12.5%) had TEC, while 28 patients (87.5%) were TEC-negative. The Phi correlation coefficient of TEC under low intensity and self-reporting photosensitivity was 0.67, and the TEC under high intensity and self-reporting photosensitivity was 0.77.

For the analysis of the associated factors, we used the TEC test result from high-intensity light. Two out of 27 TEC-positive patients (7.4%) reported diplopia, while 4 out of 34 TEC-negative patients (11.8%) reported diplopia (*p* = 0.57). The motor alignment was related to the TEC; the smaller angle of deviation at near target was associated with the presence of TEC with statistical significance (*p =* 0.04). The control scale was not a factor that affected TEC (*p* = 0.84). Sensory status was also related to the presence of TEC; normal sensory status at distance was associated with TEC with statistical significance (*p* <  0.01). Detailed statistics of TEC and photosensitivity are shown in Table 1. There were no pupil dynamics factors that significantly affected TEC (Table 2).

**Table 2:** Pupil dynamics and transient eye closure (TEC) using a near infrared vision monitoring system

Dynamics of pupil		Transient eye closure (−) (mean ± SD)	Transient eye closure (+) (mean ± SD)	*P*-value
Initial diameter (mm)	R	5.26 (±0.81)	5.71 (±0.27)	0.14
	L	5.35 (±0.71)	5.69 (±0.22)	0.18
Amplitude of contraction (mm)	R	1.96 (±0.30)	2.21 (±0.30)	0.90
	L	2.09 (±0.24)	2.18 (±0.27)	0.41
Latency of contraction (ms)	R	193.6 (±35.47)	198.3 (±74.15)	0.85
	L	191.0 (±35.81)	202.3 (±75.45)	0.66
Duration of contraction (ms)	R	642.9 (±65.47)	651.2 (±108.44)	0.83
	L	681.6 (±84.35)	670.3 (±87.74)	0.76
Velocity of contraction (mm/s)	R	6.98 (±1.96)	7.33 (±1.32)	0.63
	L	7.20 (±1.05)	7.77 (±2.51)	0.50
Latency of dilation (ms)	R	836.5 (±78.86)	849.5 (±54.80)	0.66
	L	881.6 (±72.72)	862.8 (±65.91)	0.53
Duration of dilation (ms)	R	1630.9 (±72.72)	1624.5 (±58.06)	0.82
	L	1586.2 (±79.82)	1624.7 (±44.37)	0.20
Velocity of dilation (mm/s)	R	2.63 (±1.10)	3.00 (±0.80)	0.38
	L	2.52 (±0.59)	3.00 (±0.70)	0.13

*R* Right eye, *L* Left eye, *SD* Standard deviation

Using binomial logistic regression test, multivariate analysis showed that normal sensory status at a distance was significantly associated with TEC (*p* = 0.02, Table 1) among the parameters (*p* <  0.1). The R square of the multivariate analysis was 0.61.

## Discussion

In our study, 44.3% of intermittent exotropia patients had transient eye closure with bright light. Even the TEC under low-intensity light had Phi correlation coefficient of 0.67 with self-reporting photosensitivity, while some of the patients had different responses to TEC test. The amount of exodevaition, stereopsis, the presence of diplopia, and pupil dynamic had no significance with TEC’s presence except normal sensory status at a distance in the patients with IXT.

Lew et al. suggested that photosensitivity and eye closure were more likely to occur in patients with a distance angle of strabismus > 25 prism diopters. Moreover, the study revealed that these phenomena were found to disappear after strabismus surgery, even when the surgery was deemed under-corrected [4]. However, in the study by Oh et al., the preoperative angle of deviation was not significantly different between those with a presence of squinting and those without squinting [6]. In the previous study, the term ‘squinting’ has been used as the same meaning as ‘photophobia.’ Our present study found that, by individual analysis, the smaller angle of deviation at near was associated with the presence of TEC and the distance angle was not significantly associated with the presence of TEC, even though the motor status was not significant in multivariate analysis. A previous study recruited patients who underwent surgical correction while our study involved all patients with IXT who came to our clinic without any treatment [4]. These discrepancies may be related to selection bias, as patients who required surgical treatment might have had more severe symptoms.

A study analyzing 162 patients who underwent surgical correction also showed that squinting and photosensitivity were more likely to occur in patients with stereopsis worse than 60 s [4]. However, Oh et al. revealed that stereopsis was not significantly associated with photosensitivity [6]. Our study also demonstrated that the degree of stereopsis was not related to the presence of TEC evoked by bright light.

The iris is the primary organs controlling retinal luminance, and abnormal pupil dynamics may cause photosensitivity. Dulop reported that there was abnormal pupil dynamic in patients with IXT; about one-third of patients with IXT have paradoxical pupil changes, and in these patients, pupil dilation occurs when the eyes are aligned immediately prior to exotropia [14]. However, our study revealed that pupil dynamics, pupil size, and response to light stimulation were within normal ranges. In addition, there was no significant association between pupil dynamics and the presence of transient eye closure evoked by bright light.

The presence of TEC evoked by bright light intensity showed a strong correlation with photosensitivity. Taken together, this study suggests that TEC may be a part of the light-modulating mechanism and an avoidance mechanism of photosensitivity. Therefore, we propose that TEC under bright light is a form of photic blink reflex. A photosensitivity grading study using a synoptophore, which measured subjective discomfort on a numeric scale, reported that the binocular photophobia threshold was significantly lower in participants reporting eye closure compared with those who did not [7]. Campos et al. reported that a deterioration of fusional amplitude and a weakening of binocular sensory status were shown in patients with IXT during light exposure [15]. They suggested that since bright light lowered the threshold of binocular photosensitivity, this impairment, although not conscious, may determine a subjective disturbance. To avoid this inconvenience, the patient may close one or both eyes, thereby eliminating any binocular demand [15]. However, the study was performed by comparing the IXT group to a control group, which involved people with orthophoria, esophoria, and exophoria. Some IXT patients may have more deteriorated sensory status than normal people, and therefore, the IXT group investigations may show us more significant factors related to TEC. Our study demonstrated that normal sensory status was the only significant factor in the presence of TEC in patients with IXT. These findings suggest that the TEC is independent of motor alignment, stereopsis, and pupil reflex pathway in patients with IXT. We suppose that the different pathway, such as neural circuits in the brainstem may play a role in TEC. In addition, our study demonstrated that test also provides data regarding TEC under different light intensity conditions; therefore, it may help grade the TEC in IXT patients and be an analytic tool when objective comparison of TEC is required.

From a practical clinical standpoint, our new test method helps identify the presence of TEC in patients with IXT. Near-infrared camera vision monitor system is noninvasive test method that use a Ganzfeld (Entire field) environment with a stimulator. A near-infrared camera vision monitor system can monitor pupil changes under different light intensities and observe eyelid movement simultaneously with pupil changes. This test method is easy to perform, even in children, without patient discomfort. The presence of TEC had a strong agreement with self-reporting photosensitivity in IXT patients. Although we could not investigate the physiologic mechanism between transient eye closure and photosensitivity, the evaluation of TEC using a near-infrared camera vision monitor system can be an objective and repeatable test method to assess photosensitivity in patients with IXT.

Our method has some limitations. First, it is a retrospective analysis and there could be a selection bias. However, we believe the chance of selection bias is small since all the patients visited clinics were included as source population. In second, we made up the questionnaire for the evaluation of subjective photosensitivity, and it was not validated by test-retest. However, the questionnaire included major symptoms of photosensitivity, and we believe that it is relevant for the study. Test-retest should be done in the future study. Thirdly, we did not complete the method with a normal control group to find an association of TEC and IXT, and further study is necessary. Fourth, we could not calibrate the light source’s intensity, although there is a possibility that each patient has a different TEC threshold. Further research should be conducted to find the customized luminance intensity that accounts for more precise results. Fifth, subgroup analysis was not performed. The TEC-positive group’s subgroup analysis without photosensitivity and the TEC-negative group with photosensitivity would provide us further information about this rather contradictive phenomenon. Sixth, possible correlation of TEC measured with or without glasses were not considered. There might be a glaring effect of glasses which might increase TEC. Further study should be done on the effect of glasses. Seventh, our TEC test was performed in the Ganzfeld environment, which might have caused the difference in the results compared to the real world. Finally, although the Ganzfield based infrared camera may not be easy to equip in each clinic, we expect to get help from the recent technologic development of virtual reality and head-mounted display.

In conclusion, this new test method using a near-infrared vision monitoring system with different light intensity helped evaluate the presence of TEC. The presence of TEC had an statistically significant agreement with photosensitivity in patients with IXT. In addition, our study suggested that the TEC in patients with IXT is related to photic blink reflex, which modulated independently with the angle of deviation, subnormal sensory status, and pupil dynamics.

## Supplementary Information

**Additional file 1.** Questionnaire for photosensitivity.

## Data Availability

The datasets used and/or analyzed during the current study available from the corresponding author on reasonable request.

## Abbreviations

TEC: Transient eye closure
IXT: Intermittent exotropia
CS: Control scale
W4D: Worth’s four dot test
PACT: Prism alternate cover test
PD: Prism diopter
