# Supplementary material for: Investigation of transient eye closure evoked with bright light in the patients with intermittent exotropia
Source: BMC Ophthalmol. 2021 Jul 31;21:291. doi: 10.1186/s12886-021-02046-7 (PMC8325830; doi:10.1186/s12886-021-02046-7)
Supplement: Supplementary file 1 — Additional file 1. Questionnaire for photosensitivity. [file 12886_2021_2046_MOESM1_ESM.docx]

Supplement. Questionnaire for photosensitivity.

| Questionnaires (3 out of 5 'yes') | Yes | No |
| --- | --- | --- |
| (1) Eye closure when you go out in the light |  |  |
| (2) Eye closure when you suddenly turn on the light indoors |  |  |
| (3) Say 'my eyes hurt' or 'it's dazzling' when you go out in the light |  |  |
| (4) Symptoms get better if you wear sunglasses or a hat outdoors |  |  |
| (5) You don't close your eye indoors (only when you go in sunlight) |  |  |
